# Effect of Selected Luminescent Layers on CCT, CRI, and Response Times

**DOI:** 10.3390/ma12132095

**Published:** 2019-06-28

**Authors:** Jan Jargus, Jan Vitasek, Jan Nedoma, Vladimir Vasinek, Radek Martinek

**Affiliations:** 1 Department of Telecommunications, Faculty of Electrical Engineering and Computer Science, VSB-Technical University of Ostrava, 17. listopadu 15, 708 33 Ostrava-Poruba, Czech Republic; 2 Department of Cybernetics and Biomedical Engineering, Faculty of Electrical Engineering and Computer Science, VSB-Technical University of Ostrava, 17. listopadu 15, 708 33 Ostrava-Poruba, Czech Republic

**Keywords:** visible light communication, color rendering index value, correlated color temperature

## Abstract

Phosphors have been used as wavelength converters in illumination for many years. When it is excited with blue light, the frequently used yttrium aluminium garnet doped with cerium (YAG:Ce) phosphor converts a part of blue light to a wideband yellow light, resulting in the generated light having a white color. By combining an appropriate concentration of the YAG:Ce phosphor and blue excitant light, white light of a desired correlated color temperature (CCT) can be obtained. However, this type of illumination has a lower color rendering index value (CRI). In an attempt to improve the CRI value, we mixed the YAG:Ce phosphor with europium-doped calcium sulfide phosphor (CaS:Eu), which resulted in a considerably increased CRI value. This article examines an experiment with luminescent layers consisting of a mixture of selected phosphors and polydimethylsiloxane (PDMS). Different thicknesses in these layers were achieved by changing the speed of rotation during their accumulation onto laboratory glass using the method of spin coating. The spectral characteristics of these luminescent layers as they were excited with blue light emitting diode (LED) and laser diode (LD) were then determined. A suitable combination of the YAG:Ce phosphor with a phosphor containing europium, as it was excited with a blue LED, yielded a source of white light with a CRI value of greater than 85. The response time in the tested luminescent layers to a rectangular excitant impulse (generated by a signal generator and transmitted by LD) was also measured in order to examine their potential use in visible light communications (VLC).

## 1. Introduction

White light plays a substantial role in lighting applications, two basic principles being known and applied in its practical creation. One of them is additive mixing of basic colors (red, green, and blue, i.e., RGB), and the second uses blue excitant light and a phosphor [1]. Some of the methods of producing white light using additive mixing are described; for example, in [2,3,4]. Producing white light with phosphors, i.e., on wavelength converters, has been more commonly used for many years, however. One of the best-known phosphorus used for this purpose is yttrium aluminium garnet doped with cerium (YAG:Ce). When it is excited with blue light, some of the light is converted into the longer wavelength yellow spectral component, which, combined with the non-absorbed blue spectral component, produces light perceived as white [1,5].

Many publications examine the topic of white LEDs (WLEDs) based on the YAG:Ce phosphor. For example, publications [6,7] study different ways of producing the YAG:Ce phosphor that influence the properties of the generated white light. The paper [6] describes a novel yellow phosphor with core–shell structure prepared via coating Y_2_O_3_:Ce film on an Al_2_O_3_ particle, and in paper [7] another method is reported which potentiates the spectra of YAG:Ce phosphors using organic–inorganic halide perovskites materials to enrich the red light emission region. Publications [8,9] discuss a similar topic, studying the effect of the size and shape of particles of the YAG:Ce phosphor on the resulting parameters of white light. The problems of WLEDs based on the monocrystal of YAG:Ce are discussed in detail in [10,11,12]. In the practical application of certain lighting, an important issue is the host matter of the YAG:Ce phosphor. In recent years, the host matter has often been glass, which, in combination with a phosphor, forms a solid layer of phosphor in the glass (PiG) [13,14,15]. The application potential of other flexible host matters has also been investigated. One potential host matter variant is polydimethylsiloxane (PDMS). Some options for combining the YAG:Ce phosphor and PDMS are presented, for example, in [16,17,18]. PDMS is a thermally and chemically resistant material, also reflected in the stability of the resulting layer of the YAG:Ce phosphor combined with PDMS. An extensive study of the stability of combining the YAG:Ce phosphor and PDMS, including the long-term effect of high temperatures (around 300 °C) and high optical intensities (around 150 W), is described in [19].

**Figure 1:**
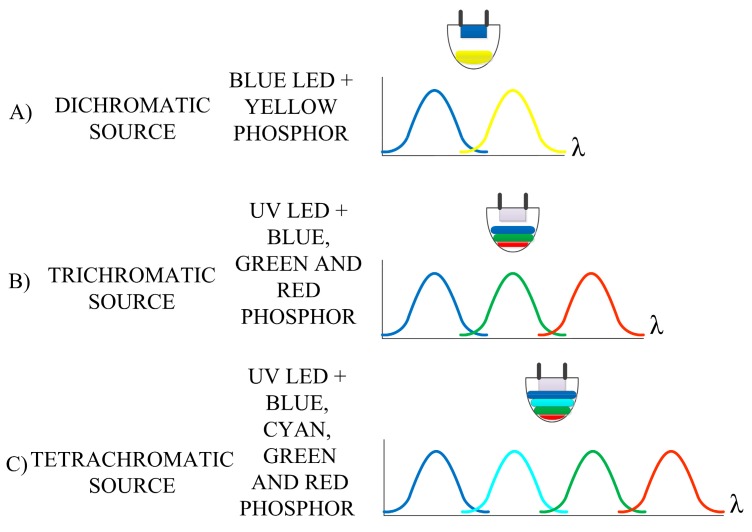
Selected types of white light sources based on wave converters.

Generating white light using a semiconductor LED and a phosphor is a viable and routine method of producing white light for general lighting applications. Several different methods create white light based on phosphors excited with LEDs. Some of these are shown in Figure 1. Generally, they can be classified as dichromatic, trichromatic, and tetrachromatic. In employing these methods, either a UV exciting source or visible (usually blue) source is used. Color rendering ability is the lowest for dichromatic sources and increases with the multi-chromaticity of the source. The CRI can reach a value of almost 100 for tetrachromatic sources [1].

In the last decade, interest in using white light not only for illumination but also for transferring information through visible light communications (VLC) has been on the rise. This area includes solutions for many interrelated topics concerning, for example, the problems of light sources suitable for use in VLC [20,21,22,23]. The issue of modifying signals and selecting appropriate modulation also has an important role [24,25,26,27,28,29]. The choice of suitable detection and subsequent demodulation of signals is no less substantial [30,31,32,33,34]. Undoubtedly, the transfer parameters are fundamentally influenced by the choice of a particular phosphor. Its dynamic properties are mainly determined by measuring the rising and falling edge times in reaction to an exciting rectangular impulse and measuring the luminescence decay time [35,36,37,38].

In this paper, we deal with luminescent layers of different thicknesses based on phosphor powders and PDMS which were produced by the spin coating method. The luminescent layers were excited using both blue light emitting diode (LED) and laser diode (LD), and the resulting spectral characteristics were compared. The phosphor delay times, which affect the communication characteristics, were measured. It was found that the CaS:Eu phosphor increased the response time. It has been shown that the thickness of the luminescent layer influences both the lighting and communication parameters. These findings can be used for optimization of the luminiscent layers in the area of VLC.

## 2. Materials and Methods

**Figure 2:**
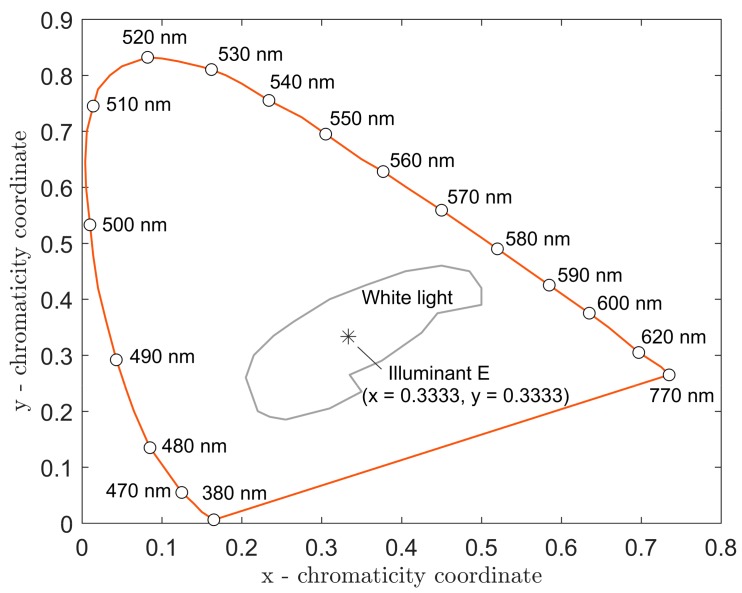
Color diagram scheme International Commission on Illumination (CIE) 1931 (*x*, *y*).

The quality of white light is assessed in the CCT and CRI parameters. The CCT is derived from the thermal radiation of a black body and associated with coordinates (*x*, *y*) in the CIE 1931 color diagram. A light source with an emission spectrum P(λ) produces tristimulus values *X*, *Y* and *Z*, which characterize the eye’s perception of color in this light source P(λ). These tristimulus values can also be used to derive the color coordinates x and y, which determine the location of the light source P(λ) in the color diagram. Clear colors are found along the contour of the chromaticity diagram. White light is located at its center (see color diagram scheme in Figure 2). The position of illuminant E is also depicted here. It is not a Planck source, but because of its spectral equilibrium, it is considered an ideal source of white light.

The known equations for calculating color coordinates from tristimulus values are shown below:

(1) $$x=\frac{X}{X+Y+Z},$$

(2) $$y=\frac{Y}{X+Y+Z},$$

(3) $$z=\frac{Z}{X+Y+Z}=1-x-y,$$

Because the color coordinate *z* can be obtained from *x* and *y*, it need not be used for the qualitative description of light [1,5].

Another important property of the white light source is its ability to accurately (truly) render the colors of physical objects, such as plants, fruits, wood, furniture, etc., that are illuminated by it. The ability to render the colors of an object is measured by the CRI (color rendering index). According to convention, it is assumed that the Planck reference source can perfectly render colors, and therefore its CRI = 100. Under international standardization, a specific set of 14 color testing patterns has been established. The first eight are used to determine the CRI. According to the CIE standard, the overall average value of the CRI is calculated as follows:

(4) $$\mathrm{CRI}=\frac{1}{8}\sum_{i=1}^{8}{\mathrm{CRI}}_{i},$$

The values ${\mathrm{CRI}}_{i}$ are calculated from:

(5) $${\mathrm{CRI}}_{i}=100-4.6\Delta E_{i}^{*},$$

where $\Delta E_{i}^{*}$ is the quantitative change of color occurring if the *i*-th testing color sample is first illuminated with the reference source and then with the tested source. Calculation of individual ${\mathrm{CRI}}_{i}$ is done so that when no color rendering dissimilarities occur, the sum of all its contributions would result in a value of 100 [39,40].

The required CCT value can be set within a wide range by changing the concentration of the YAG:Ce phosphor in the luminescent layer or changing the thickness of this layer [13,18]. However, if only the YAG:Ce phosphor is used in the luminescent layer, the CRI will reach values of around 65 to 80 only [11,15]. To obtain higher CRI values, other additives must be used in addition to the YAG:Ce phosphor. For example, in [41], a value of CRI = 83.1 is attained by adding nano-fibres emitting an orange color. Similarly, using the method of doping manganese into CsPb(Cl/Br)_3_ quantum dot glasses, a value of CRI = 81.4 was obtained [42]. Adding the red emitting phosphor Sr_2_Si_5_N_8_:Eu^2+^ and SiO_2_ particles resulted in obtaining a value of CRI = 84 [43].

After exciting the YAG:Ce phosphor with blue light, 5d→4f transitions of Ce^3+^ ions occur. These are influenced by the field of the host grid, which results in the occurrence of wideband luminescence [44,45]. The speed of these transitions is usually in the order of tens of nanoseconds [46] and affects the rising and falling edge times and the luminescence decay time [35]. These dynamic properties of phosphors affect the potential of their use in VLC and must always be determined experimentally for each particular luminescent layer [47,48].

### Preparation of Samples

A total of ten samples were prepared for the experimental measurements. Five samples contained only YAG:Ce phosphor and five samples contained a mixture of YAG:Ce and CaS:Eu phosphors (Phosphor-Technology Ltd., Stevenage, UK). PDMS of type SE 1740 (Dow Corning) was selected as the host material for these phosphors. Based on previous experimental experience, the mixture for producing these samples was prepared in two test tubes. In test tube 1, the YAG:Ce phosphor and PDMS were mixed in a weight ratio of 1:2. In test tube 2, the phosphors YAG:Ce and CaS:Eu were mixed together with PDMS in weight ratios of 1:2 and 1:20, respectively. The test tubes were then placed into a laboratory shaker for five hours to completely mix the phosphors with PDMS. Then, 0.2 g of the mixture was deposited with a pipette from test tube 1 onto each of the five laboratory glasses (samples 1–5). Similarly, 0.2 g of the mixture was deposited with a pipette from test tube 2 onto the other five laboratory glasses (samples 6–10). These laboratory glasses were than progressively placed into a WS-650Mz-23NPPB spin coater, which rotated at a speed of 400 to 600 rpm for 60 s. In this manner, a layer of different thicknesses of the PDMS and the phosphor mixture was formed on each glass. The layer’s thickness was inversely proportional to speed of rotation. At a speed of rotation of 400 rpm (samples 1 and 6), a thickness layer of more than 90 μm was formed. At a speed of rotation of 600 rpm (samples 5 and 10), a thickness layer of less than 80 μm was formed. All samples were then placed into an electric oven where they were thermally cured at a temperature of approx. 90 °C for 45 min. Layer thickness was measured with an LPT 3113i-T microscope using the method of double focusing on the upper and lower planes of the tested layers. Layer thickness was determined as the difference between the measured values of each plane. Table 1 shows the luminescent layers of respective samples 1 through 10.

## 3. Measurements and Results

**Figure 3:**
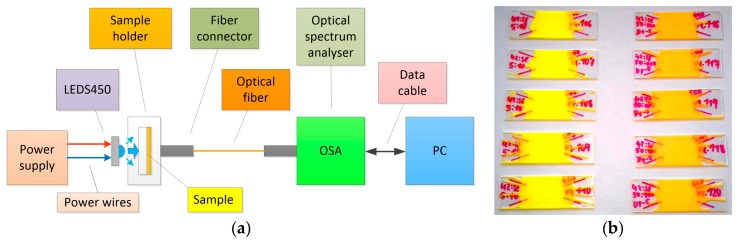
Diagram of assembly for measuring samples 1–10 (**a**), photographs of measured samples 1–10 (**b**).

Spectra were measured in all samples using an AvaSpec-HS2048XL spectrometer (Avantes BV, Apeldoorn, Netherlands). The CCT, CRI and spectra values for respective samples were recorded. The luminescent layers were measured in the central part of the samples. A blue LED (S450; Thorlabs, Newton, NJ, USA) with a dominant wavelength of 450 nm and spectral width (FWHM) of 13 nm was first used as an excitant source. The LEDS450 was powered from an HM8143 (from Rohde & Schwarz, Munich, Germany) voltage source with a working current of around 80 mA. The scheme of the measuring assembly is shown in Figure 3a, while photographs of all tested samples are shown at Figure 3b.

**Figure 4:**
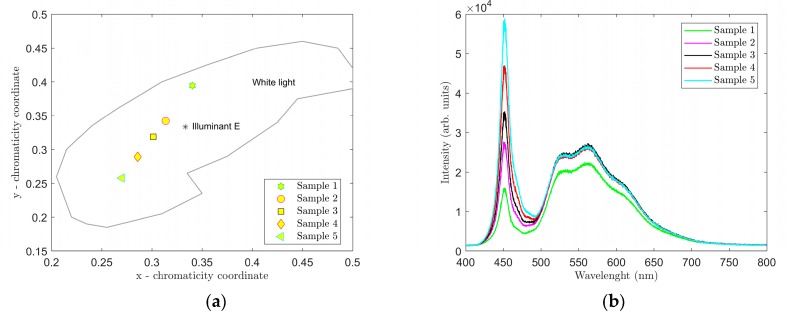
Location of samples 1–5 in the CIE 1931 color diagram (**a**), and spectra of samples 1–5 (**b**).

**Figure 5:**
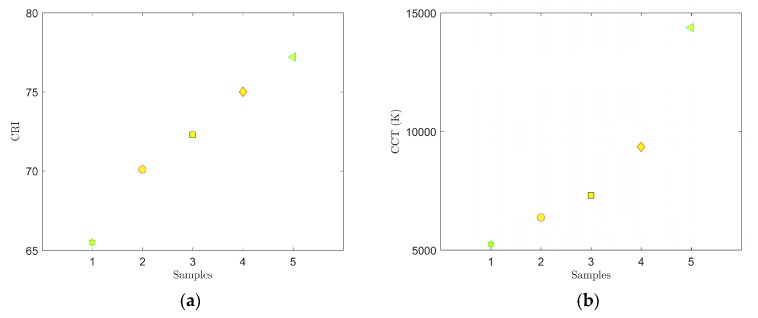
Measured CRI (**a**) and CCT (**b**) values for samples 1–5.

Spectra were first measured on samples 1–5. Data from the measurements were processed using Matlab software. The respective graphs are shown in Figure 4 and Figure 5. A summary of measured CCT and CRI values and CIE 1931 color coordinates (*x*, *y*) are presented in Table 2.

**Figure 6:**
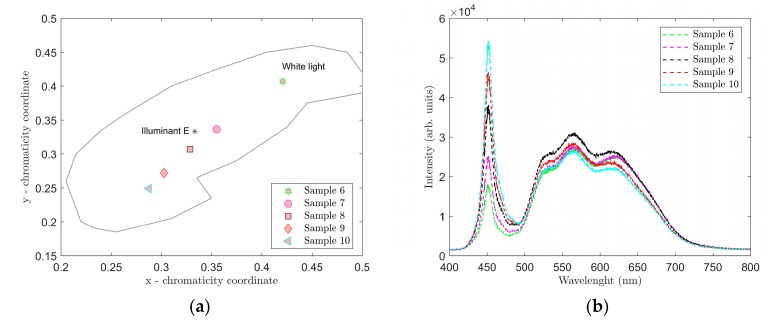
Location of samples 6–10 in CIE 1931 color diagram (**a**), and spectra of samples 6–10 (**b**).

**Figure 7:**
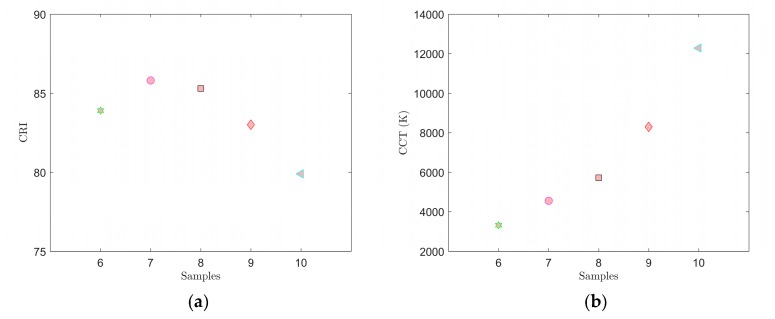
Measured CRI (**a**) and CCT (**b**) values for samples 6–10.

The results of the spectral measurements for samples 6–10 are shown in Figure 6 and Figure 7 and Table 3.

The graphs in Figure 4, Figure 5, Figure 6 and Figure 7 clearly show that parameters CCT and CRI and chromatic coordinates x, y are strongly affected by the selected phosphor (phosphors) and weight ratio to PDMS and the thickness of the luminescent film. The recorded spectra of the samples (Figure 4b and Figure 6b) clearly show the effect of thickness of the luminescent layer on the proportion of the non-absorbed blue spectral component emitted by the excitant LED. When only the YAG:Ce phosphor (samples 1–5) was used, the measured CCT values were in the range of 5250 to 14,377 K and the measured CRI values were in the range of 65.5 to 77.2 (Table 2). Figure 4 shows that the light generated by these samples falls into the area of white light; however, in samples 4 and 5, it had a strong bluish tint. Sample 2 with CCT = 6375 K and CRI = 70.1 most closely resembled standard white light (illuminant E).

Figure 6 shows the location in the color diagram, spectral characteristics and CCT and CRI values of samples 6–10. For these samples, we used a combination of the YAG:Ce and CaS:Eu phosphors that selected the red spectral component. As a result, the CRI values were considerably higher in samples 6 through 10. Samples 7 and 8 most closely resembled illuminant E, sample 7 showing CCT = 4548 K and CRI = 85.8. Adding the CaS:Eu phosphor also reduced overall the CCT values in samples 6–10 compared to samples 1 through 5. Therefore, it can be stated that the CaS:Eu phosphor caused a shift to a warmer white color.

### Measurement of Rising and Falling Edge Times

**Figure 8:**
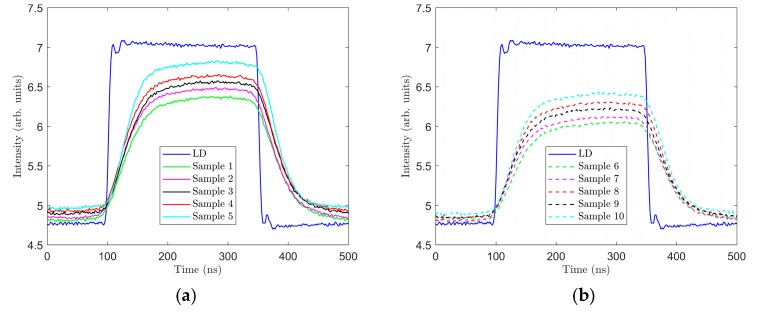
Exciting rectangular impulse and rising and falling edges in samples 1–10. (**a**) Samples 1–5; (**b**) samples 6–10.

The dynamic parameters of the phosphors used in samples 1 through 10 were also measured. These measurements were conducted with the LEDS450 (Thorlabs), which was previously used as an excitant source for spectral measurements. This LED, however, demonstrated rising and falling edge times in the order of microseconds and was therefore not suitable for this measurement. Measurement was instead conducted with laser diode (LD) PL450B (Thorlabs), which the manufacturer guarantees has a modulation speed in the order of 100 MHz. The LD was placed in an LDM38/M (Thorlabs) temperature-controlled mount and powered from the LD current controller LDC205C (Thorlabs) source. An HMF2550 signal generator was used to generate an exciting rectangular impulse frequency of f = 2 MHz (duty cycle 50%) at an LD current of 115 mA and 70 mA for level log 1 and level log 0, respectively. A PDA36A-EC (Thorlabs) photodetector and LeCroy 204Xi oscilloscope were also used. The latter equipment can automatically detect and measure the rising and falling edges. Rise time is the time during which the value of the monitored variable increases from 10% to 90%. Similarly, fall time is the time during which the value of monitored variable decreases from 90% to 10%. Figure 8 shows the recorded signal responses of the respective samples for the rectangular impulse generated by the LD PL450B. The exact results of this measurement are presented in Table 4.

It clearly follows from this measurement that rise time and fall time are strongly affected by the thickness of the measured luminescent layer and its composition. For the thickest layers (samples 1 and 6), values *t_r_* and *t_f_* were the highest, and in the thinnest layers (samples 5 and 10), values *t_r_* and *t_f_* were the lowest (Table 4). The measurements also show that the CaS:Eu phosphor contained in samples 6 through 10 increased the times *t_r_* and *t_f_* compared to samples 1 through 5 containing the YAG:Ce phosphor only. The measured values suggest that the luminescent layers with the YAG:Ce phosphor will have better communication properties for VLC than the luminescent layers containing a mixture of the YAG:Ce and CaS:Eu phosphors.

**Figure 9:**
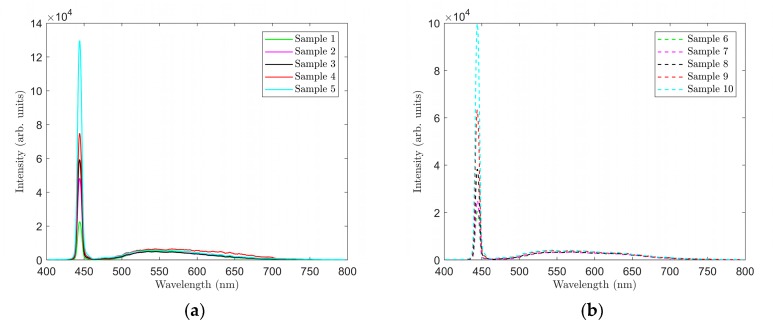
Spectra of samples 1–5 (**a**) and 6–10 (**b**).

Spectral measurements were then conducted on all samples for verification by exciting the PL450B laser diode with a working current of I = 80 mA. The measuring assembly was similar to the one used for spectral measurements with the excitant LED (Figure 3a); the difference, however, being the LDM38/M temperature-controlled mount and LDC205C current controller used to stabilize the temperature and current of the LD. The spectra recorded in this measurement are shown in Figure 9. The effect of the thickness of the luminescent layer on the proportion of the non-absorbed blue spectral component emitted by the excitant LD is once again clear.

**Figure 10:**
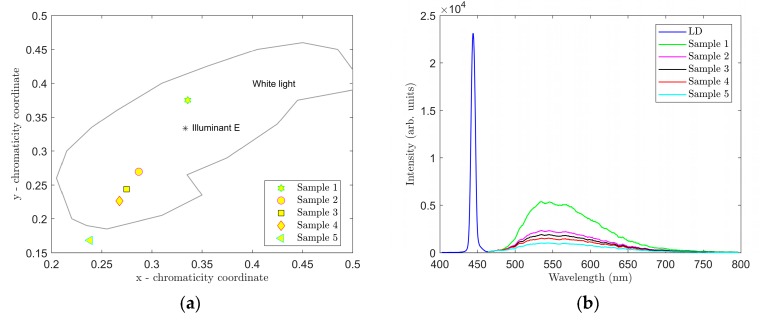
Location of samples 1–5 in the CIE 1931 color diagram (**a**), and spectra of samples 1–5 (**b**).

**Figure 11:**
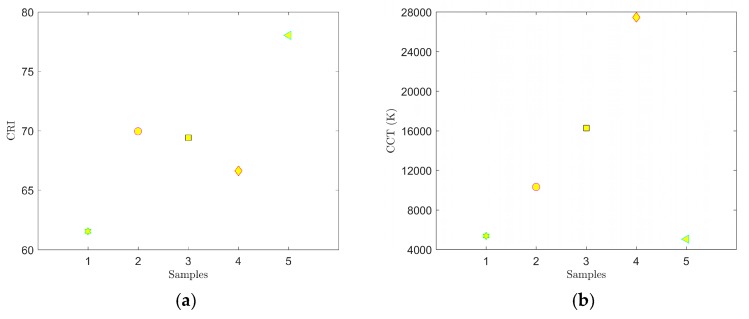
Location of samples 6–10 in the CIE 1931 color diagram (**a**), and spectra of samples 6–10 (**b**).

**Figure 12:**
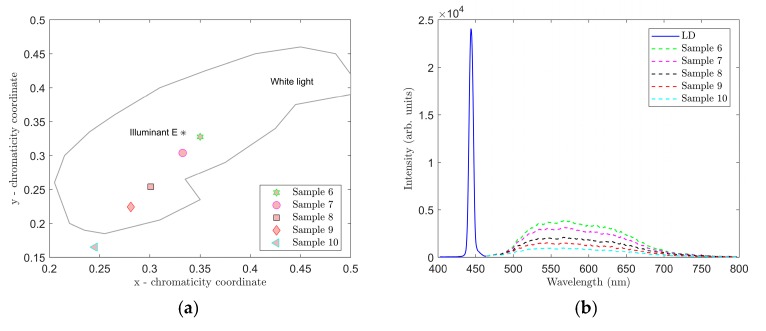
Measured CRI (**a**) and CCT (**b**) values for samples 1–5 with excitation using the LD PL450B.

The spectra in Figure 9 show that the spectral responses to LD exciting radiation in both sample groups 1 through 5 and 6 through 10 merge. Using Matlab software, the non-absorbed blue part of the spectra emitted by the excitant LD was set to the same level of intensity. As a result, the spectral responses of individual samples to this blue excitation could be satisfactorily depicted (Figure 10b and Figure 11b). The graphs from the measurement of samples 1–5 are shown in Figure 10 and Figure 12. The color coordinates, CCT, and CRI values are given in Table 5.

**Figure 13:**
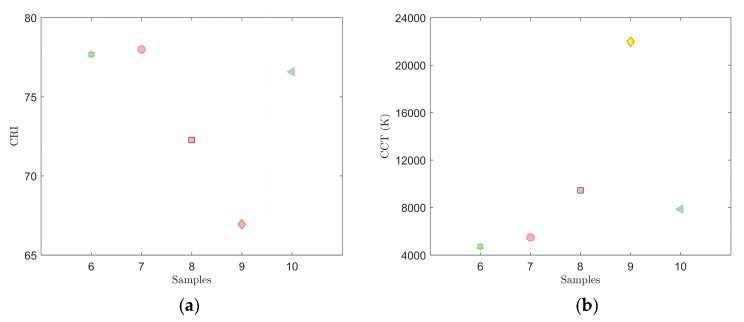
Measured CRI (**a**) and CCT (**b**) values for samples 6–10 with excitation using the LD PL450B.

The graphs of spectral measurements with excitation from the LD in samples 6–10 are shown in Figure 11 and Figure 13. The color coordinates, CCT and CRI values are in Table 6.

Figure 10 through Figure 13 and Table 5 and Table 6 clearly show that the spectral characteristics during excitation with the LD were also strongly affected by both the thickness of the tested luminescent layer and the selected phosphor and its weight ratio to PDMS. If we compare the results of spectral measurements conducted with the LED (Table 2 and Table 3) and the LD (Table 5 and Table 6), better results from the viewpoint of quality of white light were achieved in samples excited with the LED. By contrast, the LEDS450 excitant did not demonstrate that it was suitable for communication with light, whereas the LD PL450B could be modulated in the order of 100 MHz and showed that it could potentially be an appropriate source for VLC.

## 4. Conclusions

Ten luminescent samples were prepared using spin coating to deposit a thin film of a mixture of PDMS and selected phosphors on microscopic glass. The thickness of the luminescent layers of the respective samples were affected significantly by the speed of rotation during coating. In our experiments for the selected range spin coating speeds of 400 to 600 rpm showed an approximately linear indirect correlation between spin coating speeds and thickness of the luminescent layers. A combination of the YAG:Ce phosphor only and PDMS was used in samples 1–5. Samples 6–10 used a combination of YAG:Ce and CaS:Eu phosphors homogeneously distributed with PDMS. The CaS:Eu phosphor selected the red spectral component of samples 6 through 10, and when excited with the LEDS450, caused the CCT values to decrease and CRI values to increase in these samples. The color diagram in Figure 6 shows that samples 7 and 8 most closely resembled illuminant E. Sample 7 demonstrated color coordinates of CIEx = 0.3551 and CIEy = 0.3364 and values of CCT = 4548 K and CRI = 85.8. When excited with the LD PL450B, samples 6 and 7 most closely resembled illuminant E. In this case, sample 7 had color coordinates of CIEx = 0.3326 and CIEy = 0.3039 and values of CCT = 5482 K and CRI = 77.98.

Regarding communication properties, the best results were achieved in sample 5, with a rise time of *t_r_* = 58.05 ns and fall time of *t_f_* = 58.53 ns (Table 4). However, examining the spectral characteristics of sample 5 excited with the LD showed that it had a strong blue tint and was located away from area of white light (Figure 10a). The best values from the viewpoint of quality of white light were attained in sample 7 when excited with the LD, achieving values of *t_r_* = 77.77 ns and *t_f_* = 78.84 ns. This means, at these settings of the LD excitant and sample 7, that even a very short impulse generated by this assembly will be extended to time *t_p_* ≥ *t_r_* + *t_f_*, i.e., *t_p_* ≥ 156.61 ns. For sample 7 excited with the LD PL450B, this implies that its use in VLC will be adversely affected by luminescent phenomena with a frequency of more than 6.39 MHz. The experiments showed that the thickness of the luminescent layer not only strongly affected the spectral characteristics but also the dynamic characteristics of light generated by this layer. Comparing the rise and fall times (Table 4) and the spectra of samples 1–10 excited with the LD PL450B (Figure 10b and Figure 11b), we can conclude that the speed of the luminescent response (rise and fall times) was proportional to the ratio of the blue excitant and the wideband luminescent spectral component. With a greater proportion of the blue spectral component, the times *t_r_* and *t_f_* decrease, and thus communication properties would improve.

This study demonstrated that depositing luminescent layers consisting of a mixture of phosphors and PDMS by spin coating at different speeds of rotation can significantly aid in tuning the parameters of the resulting light. It was also shown that the CaS:Eu phosphor added to the luminescent layer in an appropriate weight ratio significantly increased the CRI value of the resulting light; however, it also increased rise time and fall time and therefore diminishes its potential for use in VLC. It is therefore clear that in producing luminescent layers that combine PDMS and selected phosphors, we can select whether our priority is either light or communication properties, or whether we look for a certain and reasonable compromise between them. Our future research will investigate luminescent layers of different thicknesses and different weight ratios of selected phosphors and PDMS. Our aim is to not only investigate the spectral characteristics of these layers but also their effect on communication properties with respect to certain types of modulation.

## Figures and Tables

**Table 1:** Characteristics of prepared samples 1–10.

Sample	Speed of Rotation to Prepare the Layer (rpm)	Layer Thickness (μm)	Weight Ratio YAG:Ce and PDMS	Weight Ratio CaS:Eu and PDMS
1	400	96	1:2	0
2	450	90	1:2	0
3	500	86	1:2	0
4	550	80	1:2	0
5	600	75	1:2	0
6	400	94	1:2	1:20
7	450	90	1:2	1:20
8	500	84	1:2	1:20
9	550	80	1:2	1:20
10	600	74	1:2	1:20

PDMS, polydimethylsiloxane.

**Table 2:** CCT and CRI values and color coordinates for samples 1–5.

Sample	CCT (K)	CRI (-)	*x*	*y*
1	5250	65.5	0.3403	0.3943
2	6375	70.1	0.3136	0.3421
3	7307	72.3	0.3012	0.3186
4	9350	75.0	0.2857	0.2892
5	14377	77.2	0.2699	0.2579

**Table 3:** CCT and CRI values and color coordinates for samples 6–10.

Sample	CCT (K)	CRI (-)	*x*	*y*
6	3317	83.9	0.4207	0.4070
7	4548	85.8	0.3551	0.3364
8	5724	85.3	0.3284	0.3072
9	8293	83.0	0.3026	0.2720
10	12286	79.9	0.2875	0.2490

**Table 4:** Rising and falling edge times in samples 1–10.

Sample	Rise Time *t_r_* (ns)	Fall Time *t_f_* (ns)
1	75.89	76.58
2	72.48	73.35
3	70.38	71.29
4	64.82	66.44
5	58.05	58.53
6	80.22	80.79
7	77.77	78.84
8	74.15	75.22
9	69.20	70.02
10	65.16	65.71

**Table 5:** CCT, CRI and color coordinates for samples 1–5.

Sample	CCT (K)	CRI (-)	*x*	*y*
1	5384	61.54	0.3357	0.3751
2	10328	69.96	0.2869	0.2695
3	16283	69.41	0.2747	0.2436
4	27460	66.62	0.2676	0.2261
5	5048	78.02	0.2381	0.1682

**Table 6:** CCT, CRI and color coordinates for samples 6–10.

Sample	CCT (K)	CRI (-)	*x*	*y*
6	4703	77.67	0.3500	0.3276
7	5482	77.98	0.3326	0.3039
8	9450	72.26	0.3009	0.2538
9	21956	66.94	0.2811	0.2242
10	7848	76.58	0.2453	0.1652
